# Optimizing nitrogen fertilizer via liquid bio-slurry equivalent ration for hot pepper (*Capsicum annum* L.) and soil productivity in Amhara Region, Ethiopia

**DOI:** 10.1371/journal.pone.0357581

**Published:** 2026-09-08

**Authors:** Zelalem Addis, Zelalem Ayalenh, Zimie Ambaw, Tesfaye Feyisa, Bitewelegn Kerebeh

**Affiliations:** 1 Adet Agricultural Research Center, Bahir Dar, Ethiopia; 2 Amhara Regional Agricultural Research Institute, Bahir Dar, Ethiopia; University of California Davis, UNITED STATES OF AMERICA

## Abstract

Hot pepper (Capsicum annuum L.) is a critical component of Ethiopian cuisine, widely consumed in both its green and red stages. Despite its importance, the productivity of hot pepper in the Amhara region remains suboptimal, primarily due to degraded soil fertility and the depletion of soil organic matter. To address these challenges, a two-year field experiment (2021/22–2022/23) was conducted in Jabithenan District to evaluate the impact of liquid bio-slurry (LBS) and nitrogen (N) fertilizer combinations on soil chemical properties and hot pepper yield. The study employed a randomized complete block design with six treatments: (1) control (no N), (2) recommended nitrogen (RN), (3)75% LBS + 25% RN, (4) 50% LBS + 50% RN, (5) 25% LBS + 75% RN, and (6) 100% LBS. Each treatment was replicated three times. Hot pepper seedlings (variety Mareko Fana) were transplanted into plots measuring 4.2 m × 3 m, with spacing of 0.3 m between plants and 0.7 m between rows. Soil samples were collected pre-planting and post-harvest to analyze particle size distribution, pH, total nitrogen, organic carbon, available phosphorus, and cation exchange capacity (CEC). Yield and yield components were also evaluated. Data were analyzed using ANOVA in SAS software. The results revealed that integrating LBS with RN significantly improved soil chemical properties and hot pepper yield. The 50% LBS + 50% RN and 25% LBS + 75% RN treatments exhibited superior performance, enhancing soil nutrient content and achieving higher yields compared to the control. These treatments also delivered the highest economic benefits, with a marginal rate of return exceeding 100%. Among the tested combinations, 50% LBS + 50% RN is recommended for farmers with access to sufficient LBS resources and livestock.

## 1. Introduction

Hot pepper (*Capsicum annum L.)* is one of the most important condiments in Ethiopia used both in its vegetative and matured stages in almost all dishes, especially in the form of wot (locally prepared soup).Hot pepper is a vital crop in Africa and Ethiopia due to its significant nutritional and economic value. It serves as a rich source of essential nutrients, including protein, fat, and fiber, and contains beneficial phytochemicals that enhance its dietary importance [1]. Economically, pepper is a key cash crop for smallholder farmers, playing a crucial role in poverty reduction and food security [2]. Recent studies in Ethiopia highlight that the use of improved varieties like ‘Melka Shote’ combined with optimized fertilizer application significantly boosts yield and profitability for farmers [3]. In Ethiopia, hot pepper is grown primarily for household consumption and for earning cash income [4]. It is an important cash crop in western Amhara Region particularly in Jabitahinan, Burie womberma, and Guagusa shikudad [5].The area of production in the districts increased due to its high price in the market as compared to other major food crops such as maize which is widely cultivated in the region. But its productivity in the above-mentioned areas in the region (1.67t/ha) is lower compared to the productivity in Southern Nations Nationalities and Peoples (2.008 t/ha), Tigray (1.792 t/ha) and Oromiya (1.75 t/ha) regions [6].

One of the most important yields limiting factors is soil fertility decline, which can be reversed by the use of inorganic and organic fertilizers integration (Nitrogen). Liquid bio-slurry is one of organic fertilizers that is produced from bio-digester in anaerobic condition. It contains macro nutrients N, P, K, Ca, Mg and S and micro nutrients Z, Fe, Mn and Cu which are very important for plant growth and productivity [7]. It can also contribute to soil organic matter (SOM) turnover, influencing the biological, chemical and physical soil characteristics as a soil amendment [8]. According to Warnars [9], liquid bio-slurry contains a higher level of readily available plant nutrients compared to dry bio-slurry. Because it remains in a wet and biologically active form, essential nutrients such as nitrogen (N), phosphorus (P), and potassium (K), as well as micronutrients and beneficial microorganisms, are preserved in forms that plants can absorb more efficiently [7]. In contrast, when bio-slurry is dried, a significant portion of nitrogen is lost through volatilization of ammonia, particularly during heating and long-term storage. This nutrient loss reduces the fertilizer value of dry slurry and limits its immediate effectiveness in crop nutrition.

Another advantage of liquid bio-slurry is its reduced exposure to air, which minimizes the breakdown of organic matter and loss of volatile compounds. The nutrient solution remains stable and biologically active, containing beneficial microbes that improve soil health, organic matter decomposition, and nutrient mineralization once applied to the soil. Because nutrients in liquid slurry are already partially mineralized, they are released more quickly than in dried slurry or untreated organic manure, leading to faster plant response. With all the above-mentioned advantages, organic fertilizers have some drawbacks such as slow release of nutrients, required in large quantities and have low nutrient concentration [10].

On the other hand, continuous application of inorganic fertilizers can be cause for soil fertility deterioration like soil acidity, organic matter depletion, and microbial activity & biomass decrement [11].The integrated use of inorganic and organic fertilizers can overcome the drawbacks of each fertilizer type and improve the productivity of soils [12]. However, the response of hot pepper to the integration of liquid bio-slurry and inorganic fertilizers (N) application has not been adequately studied in Amhara region. But the use of this byproduct liquid bio-slurry as organic fertilizer is little known by farmers and rate of application is not determined through research. There is also limited information about the effect of liquid bio-slurry on the soil chemical properties, especially when compared to the practice elsewhere as a soil amendment, its impacts on key parameters like pH, nutrient availability, and organic matter content remain unstudied. Current understanding relies on data from other countries with different soils and climates, making it unreliable for creating Ethiopian-specific application guidelines. This knowledge gap prevents farmers from using liquid bio-slurry effectively to improve soil health and productivity [13]. Therefore, this study was conducted with the objectives of (1) evaluating the effect of liquid bio-slurry and N fertilizer rate on soil chemical properties (2) determining the optimum rate of liquid bio-slurry and N fertilizer for production of hot pepper.

## 2. Materials and methods

### 2.1. Description of study area

**Fig 1:**
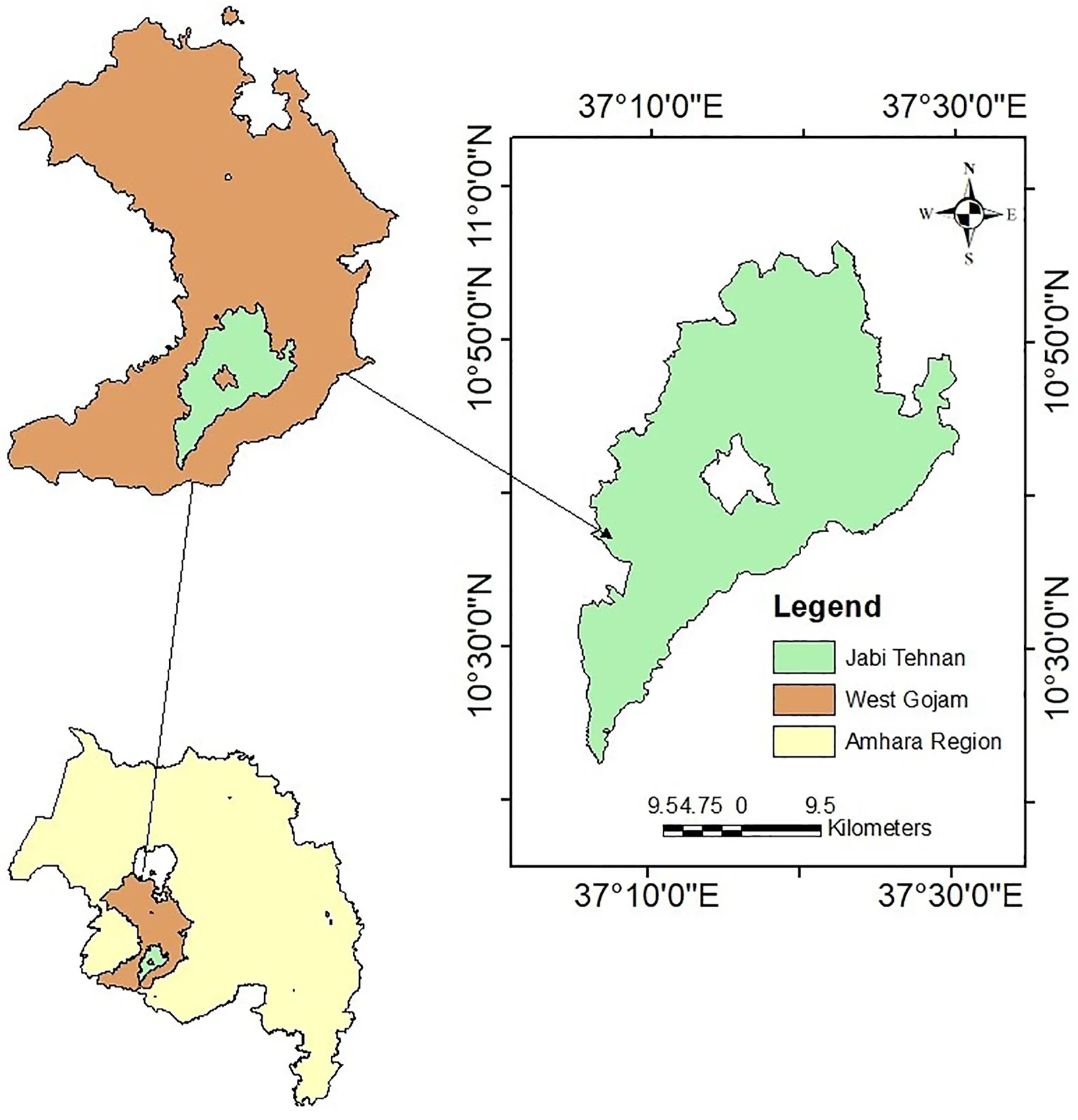
Geographical location of the study area (Jabi Tehnan District) in West Gojam Zone, Amhara Region, Ethiopia.

**Fig 2:**
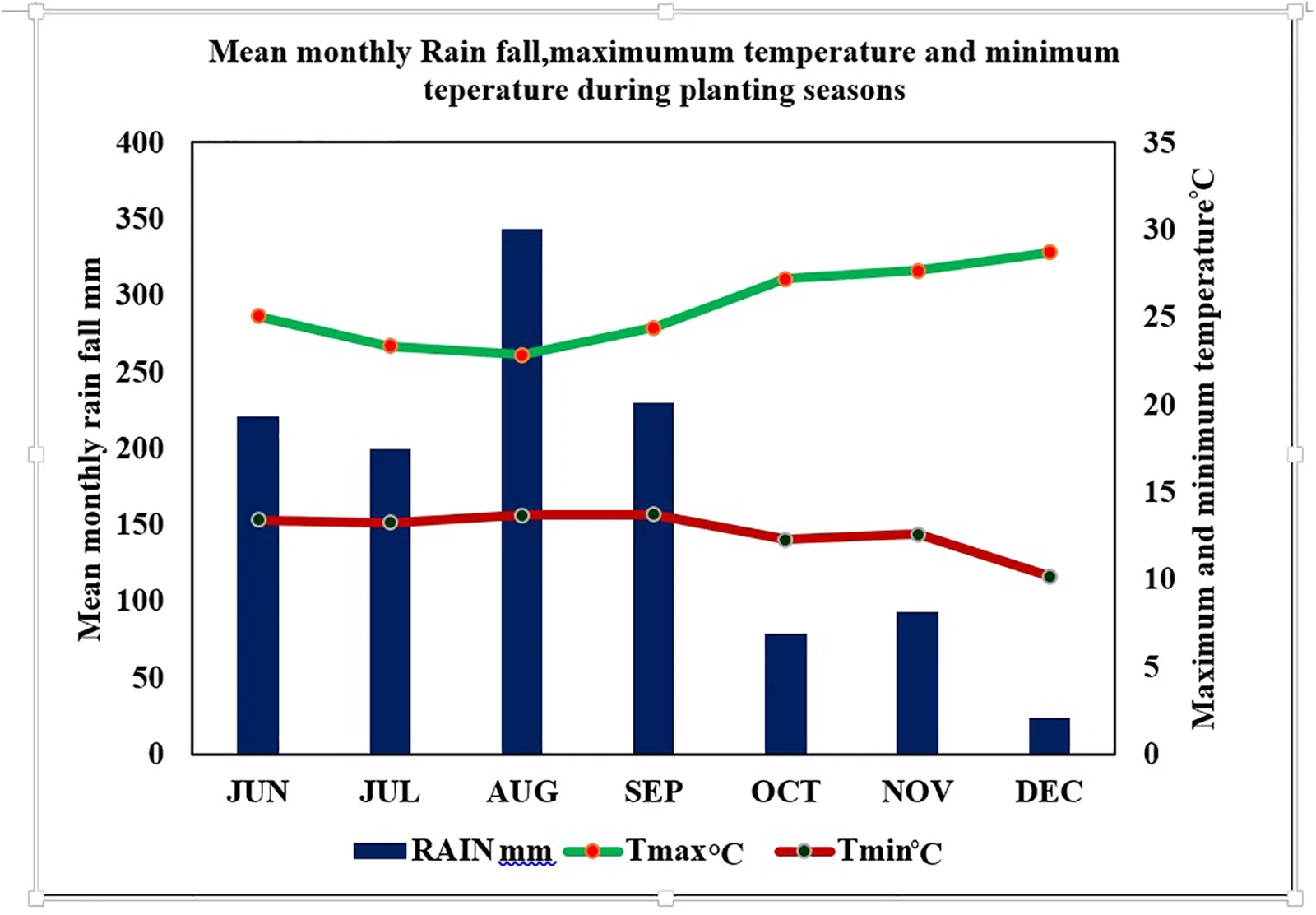
Rainfall, maximum,and minimum temperature during the cropping season based on Ethiopian Meteorological Service Agency (EMSA), North West branch, Bahir Dar. RF rainfall.

The experiment was conducted on Farmer’s field during 2021–2022 cropping seasons at Mana westegult Kebele in Jabitehenan district of West Gojam Zone in Amhara Region, Ethiopia. The study area is located 180 km south of Bahirdar. Geographically it lies at 10^0^ 44’ 13.5’‘ N and 37^0^ 10’ 26.7’‘ E (Fig 1) with a mean altitude of 2097m above sea level. It receives a mean annual rainfall of 1250 mm with mean minimum and maximum temperatures of 11.6 and 28°C, respectively (Fig 2). The landforms of the area are characterized by level plain cultivated land, scattered moderate hills, and scattered valleys [14]. Based on the district bureau of agriculture, the major land use comprises cultivated land (49.8%), natural forest (5.5%), bush and grazing land (17.6%), cultivable land (4.4%), settlement land (9.3%), and others (13.4%). Major crops, grown in the study area include Maize, Hot pepper, Finger millet, Tef, wheat, Barley, Potato, and Field pea. Soil types in the area are Nitisols, Vertisols and Luvisols. The soil of experimental site was Mollic Nitisols which is the most dominant soil type in the study area.

### 2.2. Experimental design and procedures

**Fig 3:**
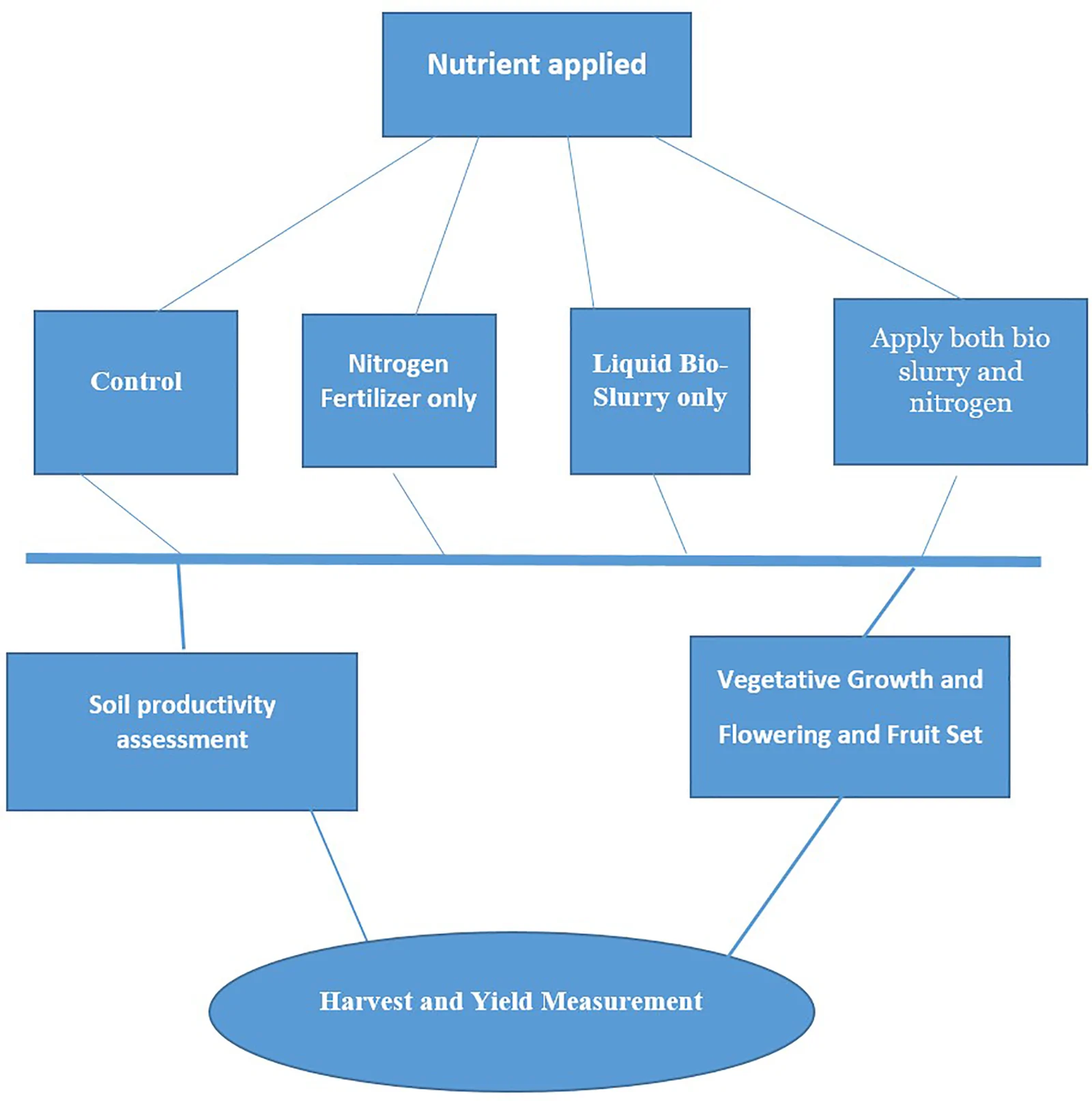
Conceptual diagram of the experimental framework illustrating nutrient management strategies and their evaluation pathway. Four treatments control, nitrogen fertilizer alone, liquid bio-slurry alone, and combined application of liquid bio-slurry and nitrogen fertilizer were applied to assess their effects on soil productivity, crop vegetative growth, flowering and fruit set, and final harvest and yield performance.

The experiment was laid out in Randomized Complete Block Design (RCBD) with three replications. Which has six treatments that include (1) control (without N), (2) recommended N (92 kg/ha N), (3) 25% N equivalent (18.625m^3^) liquid bio-slurry +75% recommended N (69 kg/ha N), (4) 50% N equivalent (37.25m^3^) liquid bio-slurry +50% recommended N (46 kg/ha N), (5) 75% N equivalent (55.875m^3^) liquid bio-slurry +25% recommended N (23 kg/ha N) and (6) 100% equivalent (74.5m^3^) liquid bio-slurry (Fig 3). The rates of liquid bio-slurry were adjusted based on recommended rate of N equivalency corresponding to its N content [21]. Urea was used as a source of recommended N whereas 92 kg P was applied in the form of TSP to all plots. The experiment was carried out under rain-fed conditions. Seedlings of hot pepper variety Mareko fana was transplanted at spacing of 0.3 m between plants and 0.7 m between rows on 12.6m^2^ (4.2 x 3 m) plot. Data were collected from the middle four rows. Space between plots and between blocks were 1 and 1.5 m respectively. Liquid bio-slurry was incorporated in to the soil one week before transplanting the seedlings of hot pepper while P was applied during the transplanting time as basal. Whereas; Urea was applied in two splits, half at transplanting and the remaining half at 50% flowering.

### 2.3. Data collection, preparation, and analysis

#### 2.3.1. Liquid- bio slurry analysis.

Liquid bio-slurry (LBS) in the tank of the bio-digester was stirred in circular movement with a stick. Preventive measure was taken to avoid scrape of the bottom and corners of the tank. Then five representative liquid bio-slurry samples were collected with a 2litter sampling plastic. Then mixed in the plastic container and a 1litter representative sample was taken for analysis of pH, organic carbon (OC), cation exchange capacity (CEC), total nitrogen (TN) and available phosphorus (AvP) by following laboratory procedures [15] in Table 1.

**Table 1:** Chemical analysis of liquid bio -slurry before incorporation.

Liquid bio-slurry chemical properties					
pH	TN (%)	CEC (cmolkg^-1^)	OC (%)	AvP (mgkg^-1^)	C: N
7.53	1.40	68.82	19.07	480	13.62

OC = organic carbon, TN = total nitrogen, C: N = carbon to nitrogen ratio, CEC = cation exchange capacity, AvP = available phosphorus, and pH = Power of hydrogen concentration.

#### 2.3.2 Soil sampling and analysis before planting.

Before planting, representative soil samples were collected from 0–20 cm depth of the experimental area in a random sampling method by using an auger. All samples were mixed together and one composite sample was formed. The composite sample was ground using a mortar and passed through a 2 mm sieve for analysis of soil texture, CEC, pH, and AvP; whereas a 0.5 mm sieve was used for determining the OC and TN. Soil pH was measured in water with the ratio of 1:2.5 using glass electrode pH meter. The soil OC content was determined following the wet digestion method as outlined by Walkley and Black which involves the digestion of soil OC with potassium dichromate (K2Cr2O7) in a sulfuric acid solution [15]. AvP was determined by Olsen extracting method. The TN content in the soil samples was determined following the Kjeldahl method. CEC also, determined by extracting the soil samples with ammonium acetate (1NNH4OAc) followed by repeated washing with ethanol (96%) to remove the excess ammonium ions in the soil solution. Percolating the NH4^+^ saturated soil with sodium chloride would displace the ammonium ions adsorbed in the soil and the ammonium liberated from the distillation was titrated using 0.1N NaOH. Wheare ass soil texture was done by particle size distribution using the hydrometer method (procedures)); sand, silt and clay percent’s were calculated and identified by using FAO textural triangle compiled by [15].

#### 2.3.1. Site description.

Results of soil chemical analysis before planting from each experimental site were presented in (Table 2). Generally, the nutrient contents of the study sites are not good in terms of availability of major plant nutrients. However, slightly acidity of a soil may be causing the fixation of AvP.

**Table 2:** Initial physicochemical properties of the experimental soil before planting.

Sites	Texture	pH	TN (%)	CEC (cmolkg^-1^)	OC (%)	AvP (mgkg^-1^)	C: N
1	Clay	5.82	0.20	28.72	1.96	16.50	9.8
2	Clay	5.08	0.17	25.72	1.72	8.50	10.1

#### 2.3.3. Crop data collection.

Crop growth and yield data were recorded at physiological maturity. For each experimental plot, five plants were randomly selected from the central rows to minimize border effects. Plant height (cm) was measured from the soil surface to the tip of the main stem using a measuring tape while the plants were still standing in the field. Pod length (cm) was determined by measuring the length of fully matured pods using a digital Vernier caliper. The number of pods per plant was counted manually from the same sampled plants, and the average value was calculated to represent a single plot reading. Dry pod yield (kg/plot) was obtained by manually harvesting all dried pods from the middle four rows of each plot to avoid edge influence. Pods were sun-dried to constant moisture content and weighed using a digital electronic balance with an accuracy of ±0.01g. All measurements and weighing were conducted under ambient laboratory conditions. The recorded values were then converted into yield per hectare according to standard agronomic procedures.

### 2.4. Economic Analysis

Economic analysis was performed to make rational choice among the applied variables in the production of hot pepper. Partial budget NB = ∑ (AB + RC) −∑ (AC + RB) where: NB = net benefit, AB = additional benefits,RC = reduced costs,AC = additional costs &RB = reduced benefits and marginal rate of return $\text{MRR}\%=\frac{\text{NBTB}-\text{NBTA}}{\text{TCTB}-\text{TCTA}}\text{X100}$ where: MRR (%) = marginal rate of return, NBTB = net benefit of treatment B, NBTA = net benefit of treatment A,TCTB = total cost of treatment B & TCTA = total cost of treatment A were used to evaluate the change in farming methods that affect partially rather than the whole farm practice and also concerned with planning tool to estimate the profit change within a farm [16]. This was computed by adjusting yield downward by a 10% and multiplying it with the local field price (240 Ethiopian Birr per kg of hot pepper). The cost of liquid bio slurry and urea was Ethiopian Birr 60 per m^3^ and 52.17 per kg, respectively. Dominance analysis was done by listing of treatments in an increasing order of cost and that has net benefit less than or equal to treatments with the lower costs that vary is dominated [16].

### 2.5. Statistical Analysis

All data were subjected to analysis of variance using general linear model (GLM) procedure by using SAS software program version 9.4 (SAS Institute, 2002). List significant test (LSD) at 0.05 probability level was employed to separate treatments means where significant differences exist [17].R-version 4.5.3 also used to perform person correlation between yield and its attributes.

## 3. Results and discussion

### 3.1. soil chemical properties before planting

The soil properties before planting revealed that the soil was slightly acidic in reaction with a pH (H_2_O 1:2.5) value range of 5.08–5.82.Which is within the range of optimum soil pH for hot pepper production [18]. The TN, CEC, OC, AvP and C: N ratio of the soil before planting were ranged 0.17–0.20%, 25.72–28.72 cmol (+) kg^-1^, 1.72–1.96%, 8.5–16.5 mg kg^-1^ and 9.8−10, respectively (Table 2). The soil total nitrogen (TN) content was classified as medium according to Olsen [18], who categorized TN concentrations of <0.10%, 0.10–0.15%, 0.15–0.25%, and >0.25% as very low, low, medium, and high, respectively. According to Tekalign [19] the soil OC content ranges of 1–2, 2–4, and 4–6% are rated as low, medium and high, respectively. While cation exchange capacity (CEC) ranges of 5–15, 15–25 and 25–40 cmol kg^-1^ are rated as low, medium and high, respectively. Based on these ratings the OC (1.72–1.96%,) and CEC (25.72–28.72 cmol (+) kg^-1^) before planting of the experimental fields were in the low and high ranges, respectively. [20] Classified AvP content of the range < 5 as very low, 5–15 as low, 15–25 as medium and > 25 mg kg^-1^ as high. Hence the AvP of the soil before planting lies under the low to high ranges.

### 3.2. Effect of liquid bio-slurry and nitrogen fertilizer on soil chemical properties

**Fig 4:**
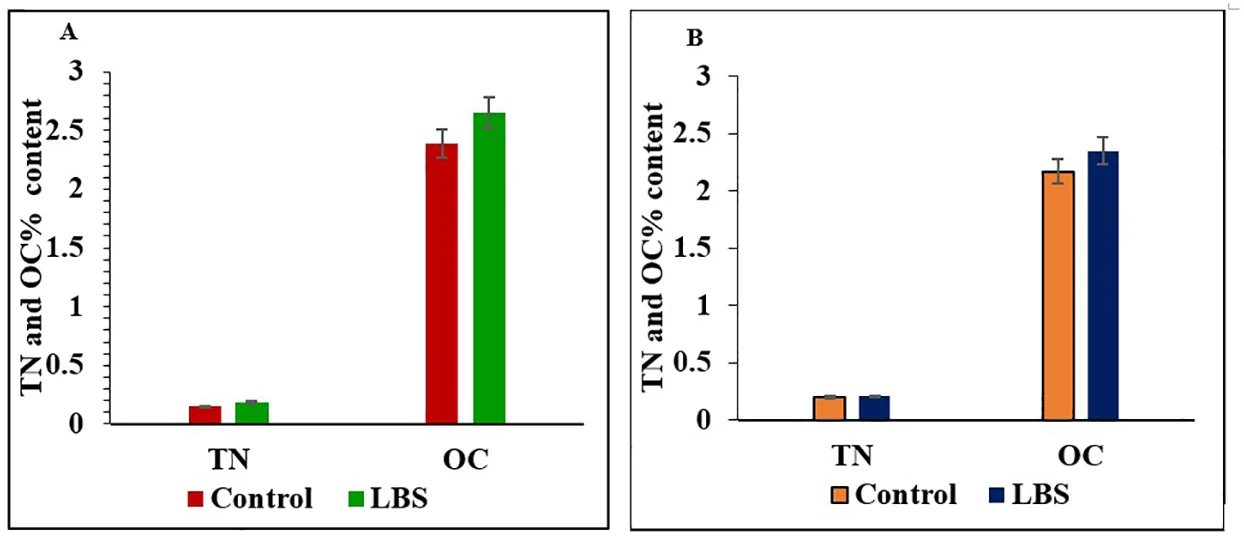
Changes in total nitrogen (TN %) and organic carbon (OC %) in plots treated with liquid bio-slurry (LBS) compared to untreated control plots at Y1S1 (A) and Y2S1 (B). Values represent mean ± standard error.

Results of soil collected after harvest indicated that integrated application of liquid bio-slurry and nitrogen fertilizer significantly (*p < 0.05*) affected soil chemical properties (Table 3). The highest values of total nitrogen (0.20%), available phosphorus (27.73 ppm) and cation exchange capacity (36.20 cmolkg^-1^) were observed by application of 100% LBS, 75% RN + 25% LBS and RN, respectively in the first year at site one (Y1S1). Similarly, the application of liquid bio-slurry and nitrogen fertilizer significantly (*p < 0.05*) affected selected soil chemical properties in second year sites (Y2S1&Y2S2). Numerically, the highest values of available phosphorus (24.52 ppm) and cation exchange capacity (35.39 cmolkg^-1^) were obtained due to application of 100% LBS, and 50% RN + 50% LBS, respectively in the second year at site one (Y2S1).On the other hand; the application of 100% LBS, and 50% RN + 50% LBS recorded higher values of pH (5.68), available phosphorus (40.36 ppm) and organic carbon (2.61%), respectively than the control in the second year at site two (Y2S2).Particularly plots treated with liquid bio slurry significantly changed total nitrogen and organic carbon contents as compared to untreated plots in Fig 4.This significant change in soil properties across sites and years might be attributed to the increasing availability of nutrients, organic matter and cation exchange capacity due to application of the liquid bio-slurry and urea. The finding agreed with Zelalem et al [21] who reported that application of 41.3m^-3^ liquid bio-slurry with 20.5kgha^-1^N significantly increased soil organic carbon compared to the untreated plots. The result is also in line with Geremew et al [22] who indicated that application of dry bio-slurry with inorganic fertilizers gave higher total nitrogen than control and recommended NP.

**Table 3:** Effects of integrated liquid bio-slurry and urea application on selected soil chemical properties at different sites over multiple years.

Seasons						
**Y1S1**	Treatments	pH	TN%	OC%	AvP (ppm)	CEC (cmol (+) kg^-1^)
	Control	5.10	0.15c	2.39	12.93b	28.79c
	RN (92kgN)	5.01	0.19ab	2.77	25.42a	36.26a
	75%RN (69kgN) +25% LBS (18.625m^3^)	5.11	0.19ab	2.48	27.73a	29.90c
	50% RN (46kgN) +50% LBS (37.25m^3^)	5.12	0.17bc	2.59	13.92b	31.13bc
	25% RN (23kgN) +75% LBS (55.875m^3^)	5.11	0.19ab	2.75	15.57b	35.04ab
	100% LBS (74.5m^3^)	5.19	0.20a	2.79	14.61b	32.77abc
		**NS**	**0.03**	**NS**	**3.1**	**4.9**
		**2.5**	**8.6**	**8.5**	**9.4**	**8.6**
**Y2S1**		pH	TN%	OC	AvP (ppm)	CEC (cmol (+) kg^-1^)
	Control	5.2	0.19	2.23	16.58bc	31.47c
	RN (92kgN)	5.01	0.20	2.40	13.75c	34.23ab
	75%RN (69kgN) +25% LBS (18.625m^3^)	5.04	0.193	2.24	17.46b	29.66c
	50% RN (46kgN) +50% LBS (37.25m^3^)	5.11	0.20	2.50	18.71b	35.39a
	25% RN (23kgN) +75% LBS (55.875m^3^)	5.13	0.20	2.55	16.19bc	31.94bc
	100% LBS (74.5m^3^)	5.10	0.20	2.60	24.52a	31.33c
		**NS**	**NS**	**NS**	**3.24**	**2.60**
		**1.7**	**3.5**	**7.9**	**10.2**	**4.5**
**Y2S2**		pH	TN%	OC	AvP (ppm)	CEC (cmol (+) kg^-1^)
	Control	5.72a	0.20	2.17b	34.90bc	33.91
	RN (92N)	5.20d	0.21	2.23b	39.82a	32.43
	75%RN (69N) +25% LBS (18.625m^3^)	5.37c	0.20	2.24b	32.74d	33.95
	50% RN (46N) +50% LBS (37.25m^3^)	5.54b	0.21	2.61a	36.58b	35.03
	25% RN+75% LBS (55.875m^3^)	5.67a	0.21	2.41ab	34.49cd	33.53
	100% LBS (74.5m^3^)	5.68a	0.21	2.15b	40.36a	33.29
**LSD**		**0.13**	**NS**	**0.29**	**1.8**	**NS**
**CV%**		**1.3**	**3.0**	**7.0**	**2.7**	**6.7**

NB: RN = recommended nitrogen, LBS = liquid bio-slurry, Y1S1=year one site one, Y2S1=year two site one, Y2S2 = year two site two, OC% = organic carbon percent, TN% = total nitrogen percent, CEC = cation exchange capacity, AvP = available phosphorus, and pH = power of hydrogen concentration

### 3.3. Integration effect of liquid bio-slurry and nitrogen fertilizers on hot pepper yields

**Fig 5:**
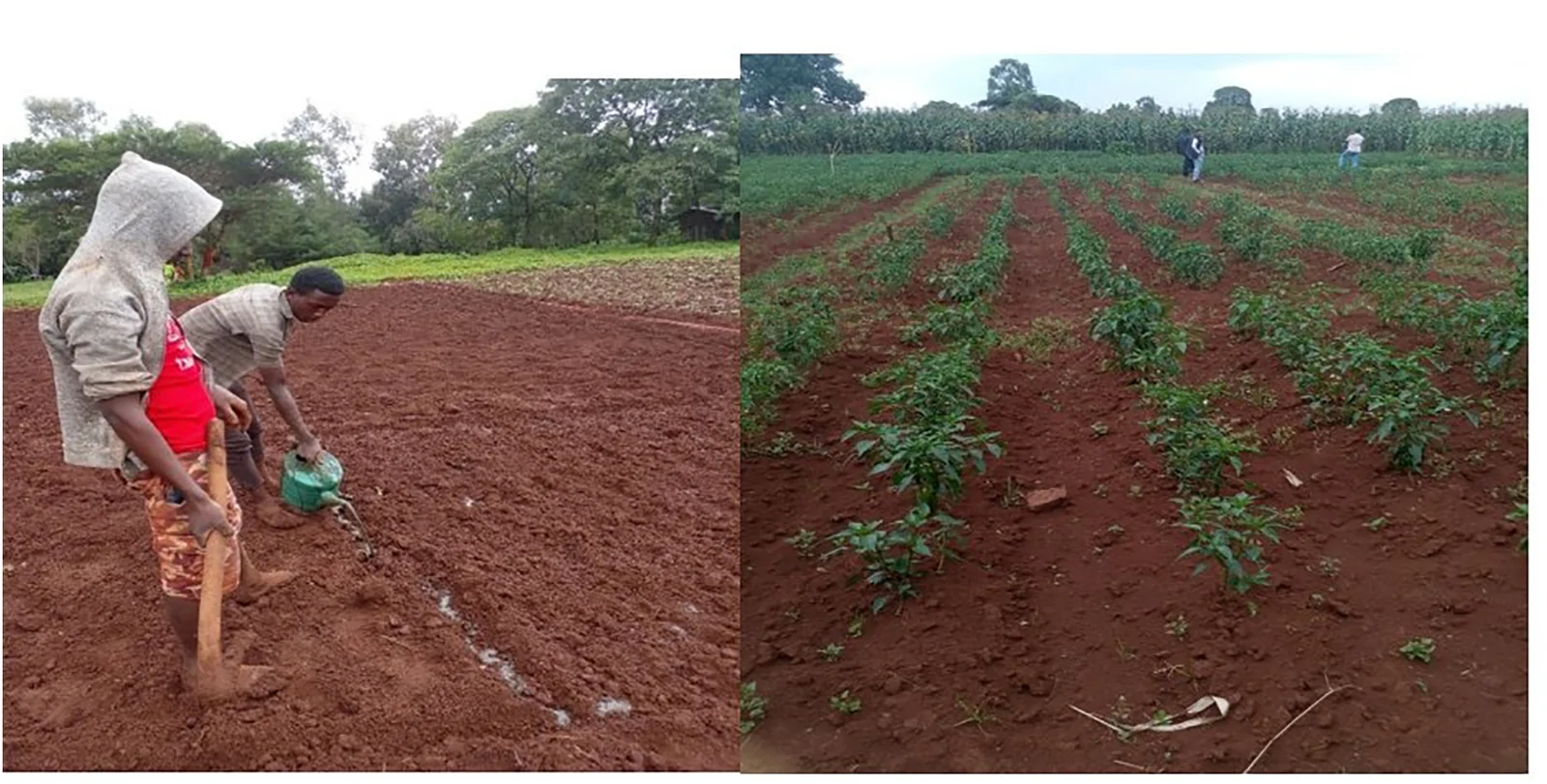
Application and soil incorporation of liquid bio-slurry and its effects on the field performance of hot pepper (*Capsicum annuum* L.), showing improved plant growth, vigor, and overall crop establishment under field conditions.

A comprehensive analysis across years and sites revealed that the yield of hot peppers significantly increased (*P < 0.05*) when LBS and N were applied in combination compared to control plots without nitrogen (Table 4). Specifically, the highest total dry pod yield (1.8 t ha ^−^ ¹) was achieved with the application of 75% recommended nitrogen (RN) combined with 25% LBS and 50% RN combined with 50% LBS. Conversely, the control plots yielded the lowest total dry pod yield at 0.97 t ha ^−^ ¹. This might be related to the synergetic effect of liquid bio-slurry and inorganic nitrogen in nitrogen uptake by hot peppers from rhizosphere. Moreover; the micro and macro nutrient contents of liquid bio-slurry may contribute to good plant growth and yield improvement (Fig 5. These findings are consistent with other studies that have explored the benefits of integrated fertilization strategies. The findings of Yalemtsehay et al [23] who reported that, the use of recommended inorganic fertilizer (100 kg DAP, 50 kg Urea and 50 kg Murate potash per hectare) with 8tha^-1^ bio-slurry gave higher yield of cabbage (266.7 tha^-1^) as compared to the yield from the control plot (160 t ha^-1^).

**Table 4:** Integrated effects of liquid bio-slurry and nitrogen fertilizer on the yield of hot pepper.

Treatment	Y1S1	Y2S1	Y2S2	^COMBINED^
	TPYtha^-1^	TPYtha^-1^	TPYtha^-1^	TPYth^-1^
Control	1.00b	0.90c	1.04c	0.97d
RN (92N)	1.80a	1.40ab	1.59ab	1.60b
75%RN (69N) +25% LBS (18.625m^3^)	1.90a	1.60a	1.84a	1.80a
50% RN (46N) +50% LBS (37.25m^3^)	1.90a	1.70a	1.72ab	1.80a
25% RN + 75% LBS (55.875m^3^)	1.70a	1.25b	1.63ab	1.50b
100% LBS (74.5m^3^)	1.28b	1.18b	1.34bc	1.26c
**LSD**	**0.3**	**0.26**	**0.39**	**0.155**
**CV%**	**9.9**	**10.9**	**14.4**	**11.0**

NB: RN = recommended nitrogen, LBS = liquid bio-slurry, Y1S1=year one site one, Y2S1=year two site one, Y2S2 = year two site two, TPY = total dry pod yield

On the other hand, the study done by Tsegaye et al [24] revealed that the lowest value of fresh shoot biomass and marketable yield of potato tuber were achieved from control while the highest values were obtained from plots treated with combined farm yard manure and recommended nitrogen. The recent research has highlighted the importance of combining organic manures like vermicompost with inorganic fertilizers to enhance nutrient availability and crop yields. A study from 2024 emphasized that integrating vermicompost with soluble fertilizers can significantly improve plant growth and soil properties, particularly when vermicompost constitutes 25% of the recommended fertilizer dosage [25].

Similarly, Javier et al [26] observed that the application of vermicompost, solarized cow manure, and solarized poultry manure resulted in higher pod yields in hot pepper plants compared to untreated controls. This study underscores the potential of organic amendments to improve crop productivity. This further supports the effectiveness of integrated nutrient management strategies. Integrated crop-livestock systems (ICLS) improve nutrient cycling and farm efficiency by repurposing animal manure as fertilizer and using crop residues for feed, thereby creating a synergistic production loop. A study from 2022 demonstrated that system fertilization in ICLS increased total herbage production and improved phosphorus and potassium use efficiency, contributing to higher economic returns compared to specialized systems [27]. Additionally, research from 2023 highlighted the importance of balancing organic and inorganic fertilizers to maintain soil health and reduce environmental impacts [28]. Moreover, a study from 2024 indicated that integrating farmyard manure (FYM) with mineral fertilizers significantly improved soil quality and maize productivity. The combination of FYM (10 t ha ^−^ ¹) with 40 kg N ha ^−^ ¹ was found to be particularly effective in achieving high sustainability yield indices in red soil under rain fed condition [29].

### 3.4. Correlation between yield and yield attribute parameters of hot pepper

**Fig 6:**
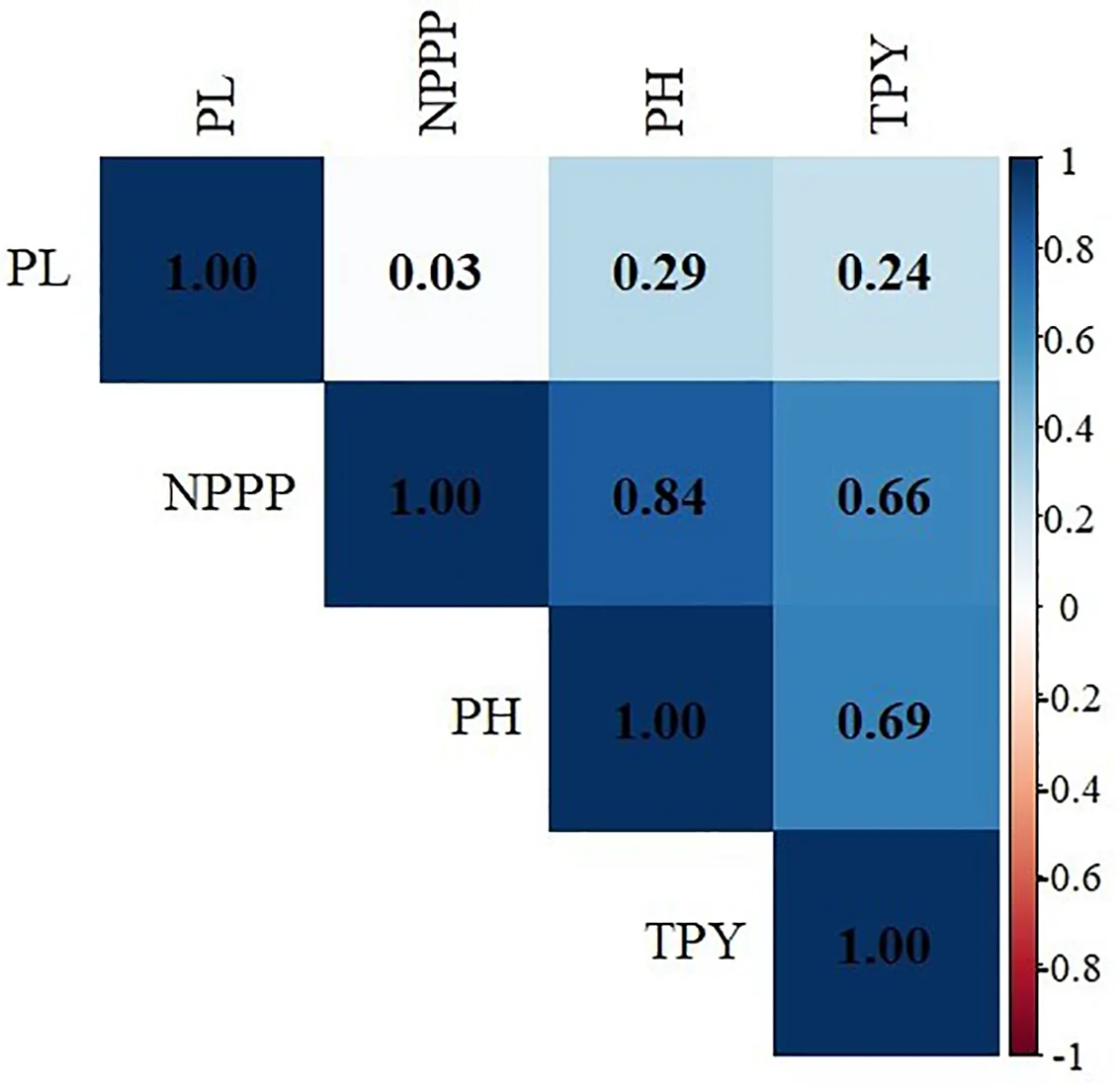
Correlations between hot pepper yield and yield-related attributes across years. NB: PL = pod length, NPPP = number of pods per plant, PH = plant height, TPY = total dry pod yield.

To elucidate the interrelationships between yield and yield-related attributes in hot pepper, a comprehensive analysis of simple correlation coefficients (r) was conducted, with results presented in (Fig 6). These coefficients provided a quantitative assessment of both the magnitude and direction of associations among the studied parameters. Notably, total dry pod yield (TPY) exhibited a robust, positive, and statistically significant correlation with plant height (0.69) and the number of pods per plant (NPPP) 0.66. This correlation suggests a substantial phenotypic linkage between these traits, implying that enhancements in either plant height or the number of pods per plant could potentially lead to improved total dry pod yield in hot pepper under field [30].

The observed positive correlation underscores the interdependence of these traits, highlighting their potential as critical targets for genetic improvement in breeding programs aimed at optimizing productivity. This finding is consistent with previous research emphasizing the importance of traits such as number of pods per plant, pod length, and pod diameter in enhancing yield [31]. These attributes have been identified as key contributors to total dry yield due to their direct and indirect effects on productivity [32]. Furthermore, path coefficient analysis has demonstrated that traits like pod length, diameter, and dry pod weight exert significant direct effects on total dry pod yield. In general, the detailed analysis of correlation coefficients highlights the importance of understanding the complex relationships between yield and yield-related traits in hot pepper.

### 3.5. Partial Budget Analysis

The transition from agronomic potential to farmer adoption hinges on economic viability, which was assessed through a partial budget analysis. This analysis focused exclusively on the variable costs that differ between treatments specifically, the costs of liquid bio-slurry (LBS), urea for the recommended nitrogen (RN), and associated labor while holding all other production costs constant. The total dry pod yield was conservatively adjusted downward by 10% to better reflect real farmers’ yields as opposed to optimized research plots [16]. The core objective was to determine if the increased yields from the integrated treatments justified their additional costs, using the marginal rate of return (MRR) as the key decision-making criterion. An MRR of 100% was established as the minimum acceptable threshold, indicating that for every one Birr invested, the farmer must receive at least one Birr back in return to justify the risk and effort. The treatment combining 50% of the recommended nitrogen with 50% LBS delivered the highest absolute net benefit (Table 5), establishing it as the most profitable option under the prevailing economic conditions of a urea price of 24 Birr kg ^−^ ¹, LBS at 60 Birr m ^−^ ³, a hot pepper field price of 240 Birr kg ^−^ ¹, and a labor cost of 70 Birr per man-day. However, a more nuanced finding was the exceptional performance of the 75% RN with 25% LBS treatment, which achieved a staggering MRR of 3328.7%. This figure signifies an extraordinary return on investment, meaning that the additional cash required to implement this specific treatment yielded back over 33 times its value in increased profits (Table 5). This provides a critical strategic choice for farmers: those with sufficient cattle and large biogas digesters can maximize their total seasonal income by adopting the 50/50 blend, while those with limited resources can still achieve near-optimal yields and phenomenal financial efficiency with the 75/25 blend, which requires less initial investment in both urea and bio-slurry. The profound implication is that the integration of LBS creates a more resilient and profitable farming system. By substituting 25–50% of purchased synthetic urea with a farm-generated resource, farmers can significantly reduce their exposure to volatile chemical fertilizer prices, lower cash expenditures, and enhance nutrient cycling within their own operations. Therefore, the recommendation for the Jabithenan district is not a single prescription, but a flexible and economically robust strategy. Farmers can confidently adopt either the 50% RN + 50% LBS or the 75% RN + 25% LBS treatment based on their specific resource endowment, secure in the knowledge that both options offer substantial financial returns and contribute to a more sustainable agricultural model.

**Table 5:** Partial budget analysis of hot pepper under the application of liquid bio-slurry and nitrogen fertilizer.

Treatments	ADPYtha^-1^	10% ADPY t ha^-1^	TVC Birr ha^-1^	NBT Birr ha^-1^	MRR%
Control	0.97	0.87	0	209373.82	–
75%RN (69 kgN) +25% LBS (18.625m^3^)	1.80	1.62	5087.5	378723.48	3328.7
50% RN (46 kgN) +50% LBS (37.25m^3^)	1.80	1.62	5255	379265.95	323.9
25% RN (23 kgN) +75% LBS (55.875m^3^)	1.53	1.35	5422.2	324936.37	D
100% LBS (74.5m^3^)	1.26	1.13	5590	266934.53	D
RN (92 kgN)	1.60	1.44	5920	339912.74	D

NB: ADPY = adjusted dry pod yield, TVC = total variable cost, NBT = net benefit, MRR = marginal rate of return

## 4. Limitations and Scope

This study focuses on the impact of integrated nitrogen fertilizer and liquid bio slurry (LBS) on soil properties and hot pepper yield in Jabitehenan district and similar agroecologies. The scope is limited to evaluating agronomic and economic outcomes, specifically changes in soil pH, organic carbon (OC), and crop yield. Findings are geographically specific and may not be directly applicable to other regions with different soil and climatic conditions. Additionally, the study excludes long-term effects, such as impacts on soil microbial health or nutrient leaching, and does not consider indirect costs like labor or environmental impacts. Economic analysis is based on direct input costs and benefits, without accounting for potential diminishing returns or long-term sustainability. Other factors affecting crop growth, such as pest control and irrigation, were assumed to be constant across treatments.

## 5. Conclusion and recommendation

Soil properties and yield of hot pepper were improved by the integrated use of liquid bio slurry with nitrogen fertilizers as compared to untreated plots. Treatments that produced higher yields without proportionally increasing the variable cost generated greater net benefits, indicating better profitability. Conversely, treatments with high input costs but only small yield improvements resulted in reduced net benefits and were therefore less economically attractive. The marginal rate of return (MRR) further demonstrated the economic efficiency of the treatments by showing the percentage return on each additional unit of investment. Treatments that provided substantial increases in net benefit relative to the added cost achieved higher MRR values, making them viable options for farmers. In contrast, treatments with low MRR values below the commonly accepted minimum threshold for profitability were found to be uneconomical. Application of 50% LBS with 50% RN resulted in a higher net benefit with 323.9% marginal return whereas, the use of 75% RN with 25% LBS could give comparable net benefit and marginal return (3328.7%). Therefore, application of 50% LBS with 50% RN should be recommended for better production of hot pepper and soil properties improvement by changing pH and OC from 5.08 to 5.54 and 1.72 to 2.61 respectively in Jabitehenan district and similar agroecology.
